# Striatal Medium-Sized Spiny Neurons: Identification by Nuclear Staining and Study of Neuronal Subpopulations in BAC Transgenic Mice

**DOI:** 10.1371/journal.pone.0004770

**Published:** 2009-03-10

**Authors:** Miriam Matamales, Jesus Bertran-Gonzalez, Lucas Salomon, Bertrand Degos, Jean-Michel Deniau, Emmanuel Valjent, Denis Hervé, Jean-Antoine Girault

**Affiliations:** 1 Inserm UMR-S 839, Paris, France; 2 Université Pierre et Marie Curie (UPMC Paris-6), Paris, France; 3 Institut du Fer à Moulin, Paris, France; 4 Inserm U667, Collège de France, Paris, France; Vrije Universiteit Amsterdam, Netherlands

## Abstract

Precise identification of neuronal populations is a major challenge in neuroscience. In the striatum, more than 95% of neurons are GABAergic medium-sized spiny neurons (MSNs), which form two intermingled populations distinguished by their projections and protein content. Those expressing dopamine D_1_-receptors (D1Rs) project preferentially to the substantia nigra pars reticulata (SNr), whereas those expressing dopamine D_2_- receptors (D2Rs) project preferentially to the lateral part of the globus pallidus (LGP). The degree of segregation of these populations has been a continuous subject of debate, and the recent introduction of bacterial artificial chromosome (BAC) transgenic mice expressing fluorescent proteins driven by specific promoters was a major progress to facilitate striatal neuron identification. However, the fraction of MSNs labeled in these mice has been recently called into question, casting doubt on the generality of results obtained with such approaches. Here, we performed an in-depth quantitative analysis of striatal neurons in *drd1a-*EGFP and *drd2-*EGFP mice. We first quantified neuronal and non-neuronal populations in the striatum, based on nuclear staining with TO-PRO-3, and immunolabeling for NeuN, DARPP-32 (dopamine- and cAMP-regulated phosphoprotein Mr∼32,000), and various markers for interneurons. TO-PRO-3 staining was sufficient to identify MSNs by their typical nuclear morphology and, with a good probability, interneuron populations. In *drd1a*-EGFP/*drd2*-EGFP double transgenic mice all MSNs expressed EGFP, which was driven in about half of them by *drd1a* promoter. Retrograde labeling showed that all MSNs projecting to the SNr expressed D1R and very few D2R (<1%). In contrast, our results were compatible with the existence of some D1R-EGFP-expressing fibers giving off terminals in the LGP. Thus, our study shows that nuclear staining is a simple method for identifying MSNs and other striatal neurons. It also unambiguously confirms the degree of segregation of MSNs in the mouse striatum and allows the full exploitation of results obtained with BAC-transgenic mice.

## Introduction

Basal ganglia form a complex neural network involved in the selection and execution of action through interactions with multiple brain areas that process sensorimotor, emotional and cognitive information [1]. The striatum is a central component of the basal ganglia that collects and processes information coming from the cerebral cortex and the thalamus [2]. Medium-sized spiny neurons (MSNs) constitute the major cell type, comprising about 95% of striatal neurons in rodents (see [3]). The remaining 5% of neurons are composed of aspiny interneurons, which have been classified on the basis of their morphology, protein content and electrophysiological properties as large cholinergic interneurons and somatostatin-, parvalbumin- and calretinin-expressing GABAergic interneurons [4].

MSNs receive excitatory glutamatergic inputs from the cerebral cortex and the thalamus, and a modulatory dopaminergic innervation from the midbrain. They belong to two intermingled subpopulations distinguished by their projections and protein expression patterns. MSNs expressing dopamine D_1_-receptors (D1Rs), dynorphin and substance P, project to the substantia nigra pars reticulata (SNr) and entopeduncular nucleus (direct striatonigral pathway), while MSNs expressing dopamine D_2_-receptors (D2Rs) and enkephalin, project to the lateral part of the globus pallidus (LGP) (indirect striatopallidal pathway) [5]–[7]. These two subpopulations are homogenously distributed throughout the striatum, and are known to have opposite behavioral effects, as they are coupled to output pathways with opposing properties [8]. However, the exact degree of segregation between these two types of MSNs has been much disputed.

The recent introduction of *drd1a*-EGFP and *drd2*-EGFP bacterial artificial chromosome (BAC) transgenic mice, in which striatonigral and striatopallidal neurons are specifically labeled [9], allowed the demonstration that subpopulations of MSNs display very distinct properties [10]–[17]. Transgenic mice with the same promoters expressing tagged DARPP-32 [18] or Cre [19] in the two populations allow further study of each cell population, and the use of other promoters [20] or different fluorescent reporters [21] provides additional powerful tools. However, the only previous study which examined whether all the MSNs express either D1R or D2R, concluded that an important fraction of MSNs (39–50%) did not express fluorescent proteins in BAC transgenic mice with *drd1a* and *drd2* promoters [21]. These results could be due to a failure to detect neurons with low expression levels of fluorescent protein. Alternatively, they could indicate the existence of a population of MSNs expressing neither D1R nor D2R. At any rate, such results cast doubt on the generality of the conclusions of all the above studies. Therefore, we undertook a study to unquestionably clarify the pattern of expression of EGFP under the control of D1R and D2R promoters in MSNs. By using a variety of markers, we validate a simple method for identifying MSNs and other striatal neurons, based on their nuclear morphology. We then show that all MSNs are labeled in double *drd1a-* and *drd2-*EGFP transgenic mice and that only neurons expressing D1R project to the SNr.

## Materials and Methods

### Animals

A total of 17 animals were used in this study. Four C57Bl/6J WT mice were used for characterization of nuclear morphology of striatal neurons and quantification of nuclei. Six C57Bl/6J–Swiss Webster hybrid transgenic mice carrying BAC that express enhanced green fluorescent protein (BAC-EGFP) under the control of D1R promoter (*drd1a*-EGFP) or D2R promoter (*drd2*-EGFP) were used for characterization of striatonigral and striatopallidal specificity of labeling. Two *drd1a*-EGFP and two *drd2*-EGFP Swiss-Webster mice were used for assessment of fluorescence intensity. Three *drd1a*-EGFP/*drd2*-EGFP double transgenic mice, each from a different crossing of *drd1a*-EGFP and *drd2*-EGFP homozygous mice, were used for quantification of fluorescent neurons. BAC-EGFP mice were originally generated by the GENSAT (Gene Expression Nervous System Atlas) program at the Rockefeller University (New York, NY) [9]. All animals were male 7- to 8-week old mice, and they were maintained in a 12 h light/dark cycle, in stable conditions of temperature (22°C) and humidity (60%), with food and water *ad libitum*. All experiments were in accordance with the guidelines of the French Agriculture and Forestry Ministry for handling animals (decree 87849, license B75-05-22).

### Retrograde tracing of striatonigral neurons

Striatonigral neurons were retrogradely labeled by unilateral injections of FluoroGold (Interchim, Montluçon, France) into the substantia nigra pars reticulata of *drd1a*- and *drd2-*EGFP mice (anteriority from bregma (mm): −3.3; laterality: 1.3, depth from cortical surface: −4.5). Animals were anaesthetized with sodium pentobarbital (60 mg/kg, i.p., Sanofi-Aventis, France), and FluoroGold (2% dissolved in 0.9% weight/vol NaCl) was injected unilaterally using a glass micropipette (tip diameter 30 µm). FluoroGold was ejected microiontophoretically by applying positive current pulses of 5 µA (5 s on/5 s off) for 20 min. Overlying skin was sutured and mice were returned to their home cages after recovery from anesthesia. Seven days after injection, animals were perfused as indicated below.

### Tissue preparation

Mice were injected with pentobarbital (500 mg/kg, i.p., Sanofi-Aventis, France) and transcardially perfused with 4% (weight/vol.) paraformaldehyde in 0.1 M sodium phosphate buffer (pH 7.5). Brains were post-fixed overnight in the same solution and stored at 4°C. Thirty-µm thick sections were cut with a vibratome (Leica, France) and stored at −20°C in a solution containing 30% (vol/vol) ethylene glycol, 30% (vol/vol) glycerol and 0.1 M sodium phosphate buffer, until they were processed for immunofluorescence. Brain regions were identified using a mouse brain atlas [22], and sections equivalent to bregma 1.18 mm (medial nucleus accumbens, dorsal striatum) and −3.16 mm (substantia nigra) were taken. Sections were processed for immunofluorescence as described below.

### Immunofluorescence

Day 1: free-floating sections were rinsed in Tris-buffered saline (TBS; 0.25 M Tris and 0.5 M NaCl, pH 7.5), incubated for 5 min in TBS containing 3% H_2_O_2_ and 10% methanol, and then rinsed three times for 10 min each in TBS. After 15 min incubation in 0.2% Triton X-100 in TBS, sections were rinsed three times in TBS again. Finally, they were incubated overnight at 4°C with the various primary antibodies. Rabbit polyclonal antibodies against fibrillarin (1∶1000, Abcam) were used as nucleolar marker. For the identification of each type of striatal neuron, monoclonal antibodies against DARPP-32 (1∶1000, gift from Paul Greengard) were used, as well as rabbit polyclonal antibodies against parvalbumin (1∶2000, Swant), calretinin (1∶2000, Swant) and choline acetyltransferase (1∶2000, Chemicon International). Rat polyclonal antibodies against somatostatin (1∶2000, Millipore Corporation) were also used. FluoroGold retrogradely marked neurons were identified using a rabbit polyclonal antibody (1∶10 000, Millipore, Molsheim, France).

Day 2: sections were rinsed three times for 10 min in TBS and incubated for 45 minutes with goat-anti mouse Alexa488-conjugated (1∶400, Molecular Probes), goat-anti mouse, rabbit or rat Cy3-coupled (1∶400, Jackson Lab) and/or goat-anti mouse or rabbit Cy5-coupled (1∶400, Jackson Lab) secondary antibodies. Sections were rinsed for 10 min twice in TBS. Eventually nuclei were counterstained by incubating for 5 min with 1 µM TO-PRO-3 iodide (Molecular Probes) diluted on TBS. Finally, sections were rinsed twice in TB (0.25 M Tris) and mounted in Vectashield Mounting Medium (Vector laboratories).

### Analysis of fluorescence

Confocal microscopy and image analysis were carried out at the *Institut du Fer à Moulin* Imaging Facility. Double- and triple-labeled images were obtained bilaterally using sequential laser scanning confocal microscopy (SP2, Leica). EGFP-labeled neurons were visualized by direct detection of endogenous fluorescence. To compare fluorescence intensity in the various transgenic strains, EGFP was recorded at three photodetection voltages (621.3v, 727.4v and 641.0v). EGFP intensity values were determined using Leica LCS software by a black-to-yellow color look-up table (LUT), in which absolute black is represented in green (RGB = 0) and absolute white is represented in blue (RGB = 255).

### Nuclear and cellular quantification

#### Classification of TO-PRO-3 stained nuclei

Sections immunolabeled with various neuronal markers were counterstained with TO-PRO-3, a double-stranded DNA intercalating fluorescent molecule, commonly used for laser confocal microscopy analysis [23], [24]. Five different categories of nuclei (A–E) were defined according to three criteria: maximum nuclear diameter, nuclear shape and heterochromatin distribution (Table 1). Using Leica LCS software, maximum nuclear diameter was defined as the longest line that crossed the nucleus. Nuclear shape was fixed by drawing the outline of the nucleus, following nuclear envelope. Heterochromatin distribution was defined regarding nuclear localization and number of densely packed regions of chromatin, highly stained with TO-PRO-3.

**Table 1:** Nuclear morphology of striatal neurons.

Neuronal marker	Category	Nuclear diameter^1^	Nuclear shape^2^	TO-PRO-3 staining^3^
DARPP-32	A	10–11 µm	Rounded	≥3 scattered clumps, sharp nuclear limit with thin discontinuous peripheral line
Parvalbumin	B	9–11 µm	Slightly elongated	1–2 central clumps
Calretinin	C	6–10 µm	Irregular rounded	1 central clump
Somatostatin	D	8–12 µm	Elliptic (axis ratio ≥1.5)	1 central clump
ChAT	E	10–13 µm	Irregular	Irregular staining with one or more clumps of various sizes

1 Nuclear diameter is defined as the longest line that can cross the nucleus.

2 Nuclear shape is fixed by drawing the outline of the nucleus.

3 Refers to regions of intense TO-PRO-3 staining (clumps). The rest of the nucleus contains light staining.

#### Validation of nuclear morphology as a marker for neuronal subtype

A total of 45 images of TO-PRO-3 stained nuclei from striatal neurons (9 for each neuronal type) were analyzed. They were mixed, randomly named and 4 unaware observers classified them into the five established categories according to criteria (A–E). Neuronal identity was then disclosed and the proportion of correct identification was determined for each category.

#### Proportion of MSNs in the striatum

The total number of cells (neuronal and non-neuronal) in striatal sections from C57Bl/6J WT mice was assessed by counting TO-PRO-3 labeled nuclei. The total number of neurons was determined by calculating the percentage of TO-PRO-3-labeled cells that were immunoreactive for the neuronal marker NeuN. The same quantification was done for DARPP-32-immunoreactive neurons and the percentage of TO-PRO-3-labeled cells that were DARPP-32-positive neurons was calculated. Next, the percentage of neurons in each nuclear staining category (A–E or X, as described above), was calculated with respect to the total number of TO-PRO-3 labeled nuclei. Finally, the percentages of neurons for each category were determined among DARPP-32-positive neurons.

#### EGFP-labeled cells in BAC-transgenic mice

All EGFP-expressing neurons were counted in 5–6 images per striatal region and classified as low-EGFP (only visible above 727.4v) and high-EGFP (detectable at 621.3v). Next, all DARPP-32-immunoreactive neurons were counted. Red and green images were then combined, and the percentages of neurons within the following categories were assessed: EGFP-negative / DARPP-32-positive; EGFP-positive / DARPP-32-positive; low-EGFP / DARPP-32-positive; high-EGFP / DARPP-32-positive neurons.

## Results

### Striatal neuronal populations display distinct nuclear morphology

**Figure 1:**
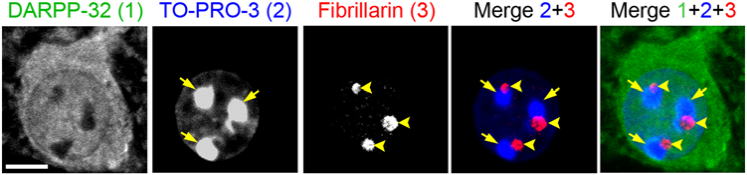
DARPP-32-positive striatal neurons have characteristic nuclear TO-PRO-3 staining. Confocal image of a striatal neuron triply stained with antibodies for DARPP-32 (green, 1), the nuclear marker TO-PRO-3 (blue, 2) and antibodies for the nucleolar protein fibrillarin (red, 3). Compact chromatin intensely stained with TO-PRO-3 (arrows) is located at the vicinity of nucleoli (arrowheads), but does not overlap with fibrillarin immunoreactivity. Scale bar: 5 µm.

Since the aim of this study was the precise quantification of neuronal populations in the striatum we first used TO-PRO-3, a fluorescent probe which labels DNA, to identify all cell nuclei. We noticed that nuclear staining with TO-PRO-3 was heterogeneous in mouse striatal sections, as in other brain regions, and we investigated whether this heterogeneity matched with known cellular types. We first characterized the staining pattern of TO-PRO-3 in cells labeled with DARPP-32, a regulatory protein found in cytoplasm and nuclei of MSNs [25], [26]. In addition to a diffuse weak and heterogeneous nuclear staining, several areas of very intense TO-PRO-3 labeling were clearly apparent within the nucleus (Fig. 1, arrows). These areas, presumably corresponding to heterochromatin, did not overlap with nucleoli, identified by fibrillarin immunofluorescence (Fig. 1, arrowheads), but were adjacent to them. Interestingly, both intensely TO-PRO-3-labeled areas and fibrillarin-positive areas coincided with virtually absent DARPP-32 immunoreactivity (Fig. 1).

**Figure 2:**
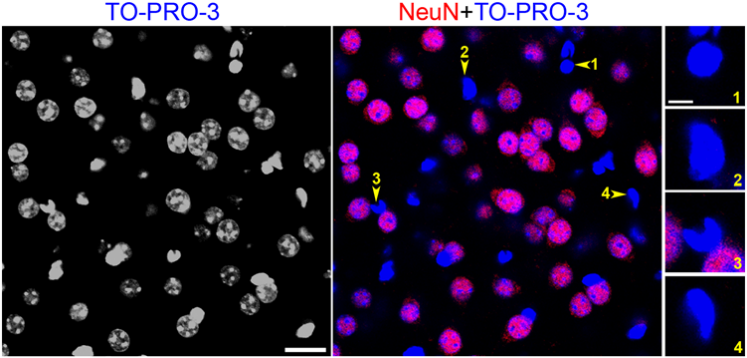
Neuronal and non-neuronal striatal cells can be distinguished by their nuclear staining. Confocal section of a striatal slice doubly stained with the pan-neuronal marker NeuN (red) and the nuclear marker TO-PRO-3 (blue). Nuclei from non-neuronal celIs are small, compact, and diffusely and intensely stained with TO-PRO-3. In contrast, NeuN-positive cells (neurons) have bigger nuclei, with diffuse irregular light TO-PRO-3 staining containing clumps of intense fluorescence. Scale bar: 20 µm. Insets on the right panels are four examples (1–4) of nuclear morphology of non-neuronal cells. Scale bar: 5 µm.

To determine whether we could distinguish neurons and non-neuronal cells on the basis of their nuclear morphology, we used antibodies for NeuN, a general neuronal marker [27]. NeuN-negative nuclei, presumably from glial cells, could easily be differentiated from those of neurons by virtue of the intense and compact TO-PRO-3 staining of the entire nucleus (Fig. 2).

**Figure 3:**
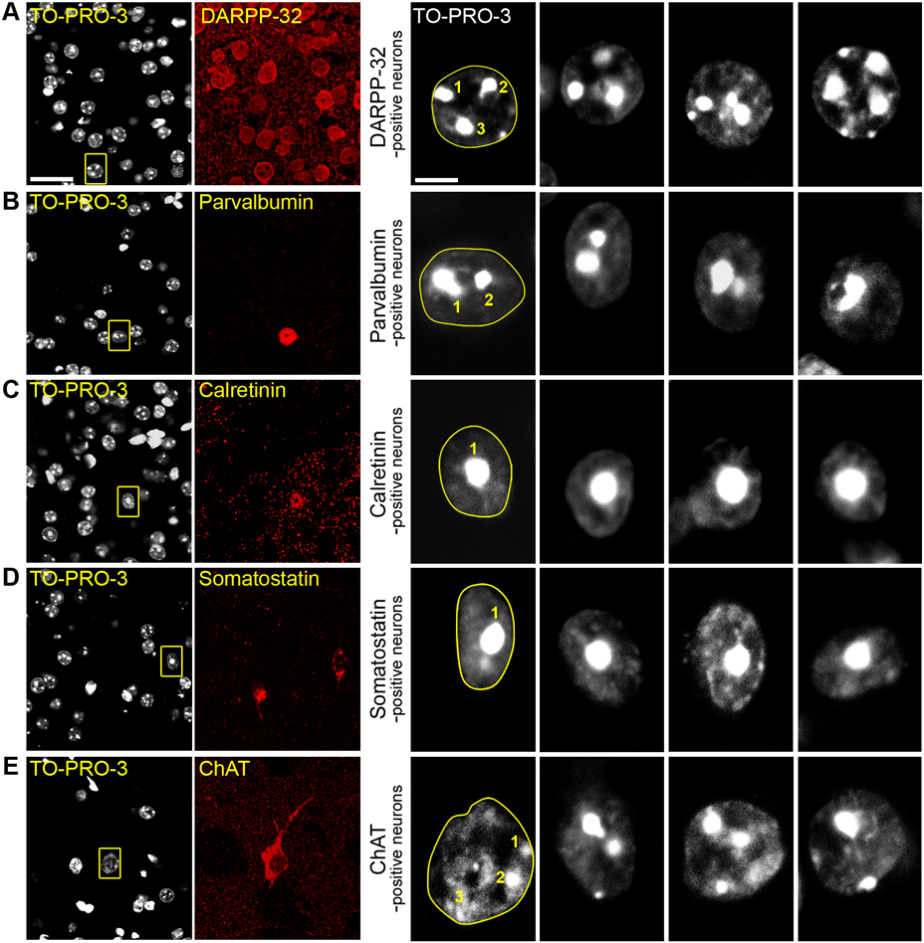
The various striatal neuronal populations have distinct nuclear morphology. Mouse striatal slices were labeled with various markers specific for each type of striatal neuron and analyzed by confocal microscopy. For each row, the left panel shows a double staining with an antibody specific for a neuronal subtype and TO-PRO-3 (scale bar: 25 µm), the right panels are high magnification of TO-PRO-3-stained neurons (scale bar: 5 µm). For the first high magnification picture, which corresponds to the boxed area in the left panel, the dashed line follows the nuclear envelope and the number of clumps of highly compacted chromatin is indicated. (A) Neurons positive for DARPP-32, a marker of medium-sized spiny neurons, have a rounded nucleus about 10–11 µm of diameter with ≥3 clumps of intense TO-PRO-3 staining. (B–D) GABAergic interneurons. (B) Parvalbumin-positive interneurons have slightly elongated nuclei of 9–11 µm of diameter. Chromatin clumps are frequently observed surrounding the nucleolus, forming 1 or 2 clusters. (C) Nuclei of calretinin-positive interneurons are small (6–10 µm), irregular but mostly circular and have a unique central clump of heterochromatin. (D) Somatostatin-positive interneurons often have elongated nuclei (longest axis 8–12 µm, axis ratio ≥1.5) with a central clump of dense chromatin. (E) Cholinergic interneurons, identified by choline acetyltransferase (ChAT) immunoreactivity, have the largest nuclei (10–13 µm) with diffuse and irregular TO-PRO-3 staining with small clumps of intense fluorescence.

We then undertook a systematic study of TO-PRO-3 labeling of the striatal neuronal populations. We doubly labeled cells with TO-PRO-3 and antibodies for proteins specific of the various neuronal subtypes: DARPP-32 for MSNs [28], choline acetyltransferase (ChAT) for cholinergic interneurons, and parvalbumin, calretinin, and somatostatin for GABAergic interneurons [4] (Fig. 3). Interestingly, nuclear diameter and shape, as well as TO-PRO-3 staining appeared to be different for each neuronal type (Fig. 3). DARPP-32-positive neurons contained rounded nuclei with a 10–11-µm diameter, characterized by the presence of 3–4 clumps of compact chromatin intensely stained with TO-PRO-3 and a thin discontinuous lining of TO-PRO-3 staining at the periphery of the nucleus, providing a sharp boundary (Fig. 1, 3A). GABAergic interneurons expressing parvalbumin had elongated nuclei with a diameter of 9 to 11 µm, in which areas of intense TO-PRO-3 labeling formed 1 or 2 clumps in the vicinity of what was likely to be the nucleolus (Fig. 3B). Calretinin-positive cells were characterized by their small size (6–10 µm) and the fact that TO-PRO-3 labeled heterochromatin was typically concentrated as a unique cluster in the central part of the nucleus (Fig. 3C). Somatostatin-containing interneurons also displayed small nuclei with heterochromatin located in a single central clump, but could be distinguished from the other types of interneurons by their elongated nuclei (axis ratio ≥1.5), in agreement with previous studies in which the fusiform perikarya of somatostatin-positive neurons was defined as a typical morphological characteristic [29] (Fig. 3D). Finally, nuclei of cholinergic interneurons were easily distinguishable from the other striatal neurons by virtue of their large size (about 12 µm, Fig. 3E). TO-PRO-3 staining was irregular with one or more clumps of various sizes.

### Nuclear morphology is a good marker for striatal neuronal types

Since TO-PRO-3 nuclear staining nuclei appeared to be systematically different among striatal neuronal subtypes, we tested whether it could be sufficient for their identification. To do so, we used criteria based on the nuclear appearance of each of the five types of striatal neurons (Table 1). We assessed the capacity of four unaware observers to identify striatal neurons on the basis of their nuclear morphology after TO-PRO-3 staining (Fig. S1). The percentage of correct identification of MSNs, and calretinin- or ChAT-positive interneurons was 90% or higher (Fig. S1). Parvalbumin- and somatostatin-positive neurons were correctly identified in 80% and 70% of the cases, respectively (Fig. S1). The parvalbumin-positive neurons were easily identified when they had 2 chromatin clumps, and the somatostatin-positive neurons when their elongated shape was apparent, i.e. when the section plane was sufficiently parallel to the longest axis. However, these conditions were not always satisfied. Altogether, our results show that nuclear morphology allows the identification of MSNs with a high success rate and is a good marker for interneuron subtypes.

### Identification of MSNs based on DARPP-32 immunoreactivity and nuclear morphology

**Figure 4:**
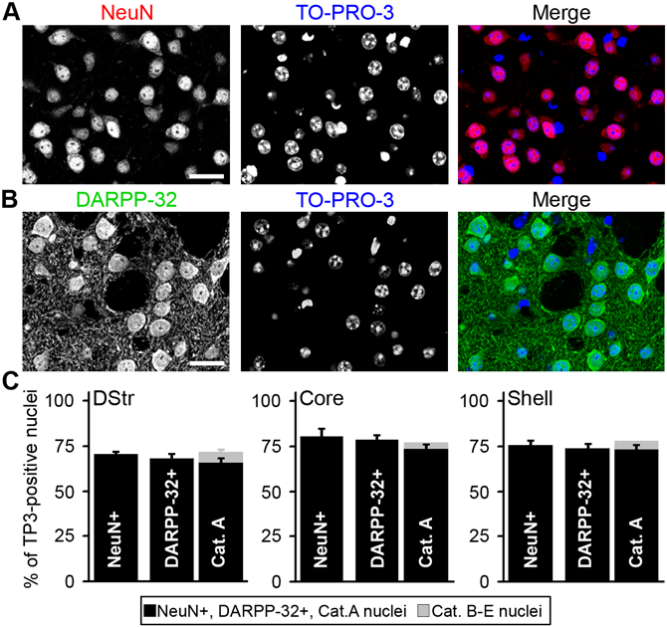
Proportion of DARPP-32-positive cells in the striatum. Low-magnification confocal section of a mouse striatal slice doubly stained with NeuN antibodies (red) and TO-PRO-3 (blue) (A), or with DARPP-32 antibodies (green) and TO-PRO-3 (blue) (B). Scale bars: 25 µm. (C) Bar graphs representing the proportion of cells identified as neurons by NeuN immunoreactivity (NeuN+), of DARPP-32-immunoreactive neurons (DARPP-32+) and of nuclei classified into A or B–E categories. The total number of cells (neuronal and non-neuronal) was determined by nuclear staining with TO-PRO-3 (TP3) in mouse dorsal striatum (DStr) and nucleus accumbens core (Core) and shell (Shell). The small percentage of cells classified in B–E categories, corresponds to striatal interneurons. Data are means±SEM; n = 3 mice; 2908 (NeuN count), 2091 (DARPP-32 count) and 1517 (nuclei categories count) cells counted.

We used the tools validated above to accurately estimate the proportion of MSNs in the striatum, taking into account both DARPP-32 immunoreactivity and nuclear morphology. We determined the total number of cells in striatal sections by double staining with the pan-neuronal marker NeuN and TO-PRO-3 (Fig. 4A), or with DARPP-32 and TO-PRO-3 (Fig. 4B). We then counted for each double staining the number of cells identified by their TO-PRO-3-stained nuclei that were NeuN-positive or DARPP-32-positive (Fig. 4C). In parallel we counted the number of neurons in categories A–E defined in Table 1. The percentage of NeuN positive cells was 70±0.4% in the dorsal striatum, 80±1.4% in the core of the nucleus accumbens (NAc), and 75±3.1% in the shell (means±SEM, 3 mice) (Fig. 4C). DARPP-32-positive neurons accounted for 68±2.3% of the cells in dorsal striatum, 78±1.4% in NAc core and 73±2% in NAc shell (Fig. 4C) (means±SEM, 3 mice). Thus, as expected, DARPP-32-positive neurons formed the largest population of striatal neurons (i.e. about 97% of the neurons in the 3 striatal regions). Similar results were obtained when classification of all nuclei was done following the five categories described previously (Fig. 4C). Note that the percentages of cells in category A was consistently slightly lower than those obtained with DARPP-32 immunolabeling because identification on the basis of nuclear morphology required a section in which the nucleus was well visible and its features identifiable, which was not the case in all DARPP-32-positive cells. However, it should be pointed out that when the nucleus of DARPP-32-positive cells was clearly visible it always had the typical morphology described in Fig. 3A. All nuclei classified in category A were DARPP-32-immunolabeled (100±0%) and virtually no DARPP-32-positive neuron was misclassified into the other categories (1±1%). Nuclei classified in categories B–E constituted approximately 5% of the total. These results combined with our previous observation that DARPP-32-positive neurons are not labeled with markers for striatal interneurons [13] demonstrate that DARPP-32 antibodies label all MSNs and only MSNs in the striatum.

### EGFP expression driven by the *drd1a* and *drd2* promoters labels all MSNs in BAC-EGFP transgenic mice

Since we had validated tools to accurately and exhaustively identify striatal MSNs, we assessed the exact proportions of DARPP-32-positive MSNs expressing the dopamine D1R and those expressing the D2R. For this purpose we used two strains of BAC transgenic mice (*drd1a*-EGFP and *drd2*-EGFP) [9] which have been extensively studied [10]–[17]. When we compared the intensity of EGFP auto-fluorescence in the two strains, we noticed that it was always weaker in *drd1a*-EGFP mice than in *drd2*-EGFP mice. Photodetection of EGFP at a low voltage did not allow a correct visualization of striatal D1R neurons in *drd1a*-EGFP animals (Fig. S2A1, 1′), while it was sufficient for identifying D2R neurons in *drd2*-EGFP mice (Fig. S2A2, 2′). A high photodetection voltage permitted a correct identification of EGFP neurons in *drd1a*-EGFP mice (Fig. S2B1, 1′), whereas it generated a saturated signal for D2R neurons in *drd2*-EGFP mice (Fig. S2B2, 2′). This marked difference in fluorescence intensity between *drd1a*-EGFP and *drd2*-EGFP strains was observed in all mice analyzed and presumably reflected differences in EGFP expression levels. We then crossed the two transgenic lines and studied the F1 progeny, which was hemizygous for both BAC transgenes. Analysis of striata of *drd1a*-EGFP/*drd2*-EGFP mice at a medium photodetection voltage, sufficient to visualize all neurons in *drd1a*-EGFP mice, showed a clear distinction between weakly labeled neurons (Fig. S2C1, 1′), which were likely to correspond to D1R-expressing neurons, and intensely labeled neurons (Fig. S2C2, 2′), which were likely to correspond to neurons expressing D2R or both receptors.

**Figure 5:**
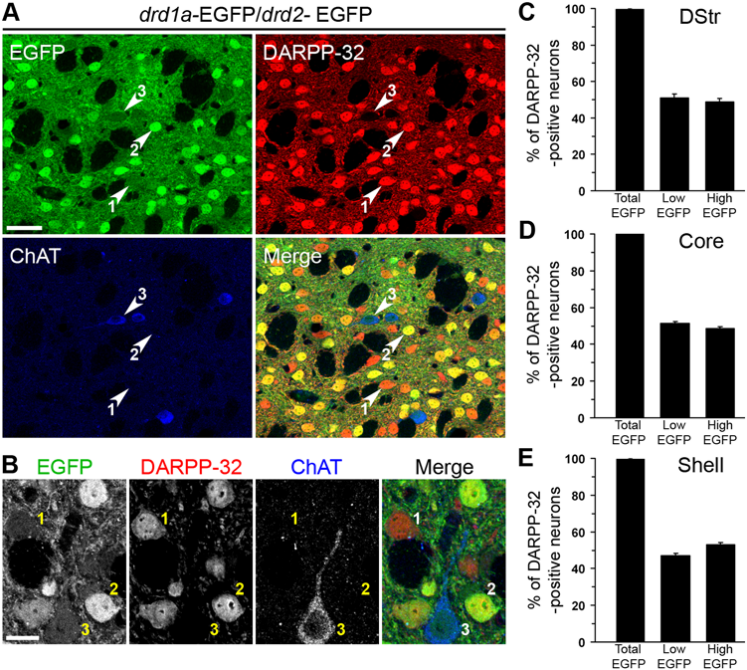
All MSNs express EGFP in *drd1a*-EGFP*/drd2*-EGFP double transgenic mice. (A, B) Striatal slices from *drd1a*-EGFP/*drd2*-EGFP mice were studied by triple labeling for DARPP-32 immunoreactivity (red), ChAT immunoreactivity (blue) and EGFP autofluorescence (green). EGFP fluorescence was classified as high or low (see Fig. S2). Note that all high-EGFP neurons are MSNs, since they are always colabeled for DARPP-32, whereas low-EGFP neurons can be either MSNs (labeled for DARPP-32) or cholinergic interneurons (labeled for ChAT). Neurons could be therefore classified into three categories: 1, low EGFP/DARPP-32-positive (D1R-expressing MSNs); 2, high-EGFP/DARPP-32 positive (D2R-expressing MSNs, possibly coexpressing D1R); and 3, low EGFP/DARPP-32-negative (cholinergic interneurons expressing D2R). (B) Higher magnification showing neurons in the three categories defined above. Images are single confocal sections. Scale bars: 40 µm (A), 10 µm (B). (C–E) The percentage of DARPP-32-immunoreactive neurons showing any (Total EGFP), low (Low-EGFP) or high (High-EGFP) EGFP fluorescence in the dorsal striatum (DStr, C), nucleus accumbens core (Core, D) and shell (Shell, E). All EGFP neurons that do not show DARPP-32 immunoreactivity were identified as cholinergic interneurons. Data are means±SEM; n = 3 mice; 967 (C), 1708 (D) and 1271 (E) cells counted.

We have previously reported that EGFP is detected only in DARPP-32-positive neurons in the striatum of *drd1a*-EGFP mice, while in *drd2*-EGFP mice it is detected in DARPP-32-positive and in a few DARPP-32-negative and ChAT-positive neurons [13]. However, our previous study did not determine whether all DARPP-32-positive cells expressed EGFP under the control of either *drd1a* or *drd2* promoter. In fact a recent report [21] concluded that a relatively large proportion of striatal neurons (39–50%) do not express EGFP in double transgenic BAC lines. To address this issue we performed a triple fluorescence analysis combining EGFP fluorescence with DARPP-32 and ChAT immunofluorescence in *drd1a*-EGFP/*drd2*-EGFP double transgenic mice (Fig. 5A, B). The majority of weakly EGFP-labeled neurons were identified as MSNs (Fig. 5A, B, type 1), as well as the totality of intense EGFP neurons (Fig. 5A, B, type 2), as revealed by their DARPP-32 immunoreactivity. The small proportion of weak EGFP neurons that were negative for DARPP-32 were always identified as cholinergic interneurons, as shown by colabeling for ChAT (Fig. 5A, B, type 3) [30].

We next examined the proportion of striatal MSNs which expressed EGFP in the striatum of *drd1a*-EGFP*/drd2*-EGFP double transgenic mice, by calculating the percentage of DARPP-32-positive cells that showed EGFP fluorescence. As shown in Fig. 5C–E, this percentage was always 100% in the various regions of the striatum (Total EGFP bar). The same results were obtained when MSNs were identified only on the basis of their nuclear morphology (data not shown). We were simply unable to find any DARPP-32-immunoreactive neuron which lacked EGFP fluorescence. These results clearly demonstrate that all striatal MSNs express either *drd1a*-EGFP or *drd2*-EGFP, or both, in double transgenic mice, in sharp contrast to what has been reported recently [21].

We then calculated the percentage of DARPP-32-immunoreactive neurons that showed a weak EGFP intensity (Fig. 5C–E, Low EGFP bar graphs) and those showing a strong EGFP intensity (Fig. 5C–E, High EGFP bar graphs). In the dorsal striatum and NAc core, the proportion of weakly labeled neurons and intensely labeled neurons was similar (about 51 and 49% respectively, Figs. 5C,D). In the NAc shell, a slightly higher percentage of intensely labeled neurons was observed (about 53%, Fig. 5E). It is important to note that weakly labeled neurons corresponded to D1R-expressing neurons, whereas intensely labeled neurons could correspond to neurons expressing either D2R alone or both D1R and D2R. If we take this into consideration, the numbers obtained here are similar to the proportions obtained in a previous study [13], in which populations were estimated from separate *drd1a*-EGFP and *drd2*-EGFP mice.

### Striatonigral neurons are virtually exclusively D1R-expressing neurons

**Figure 6:**
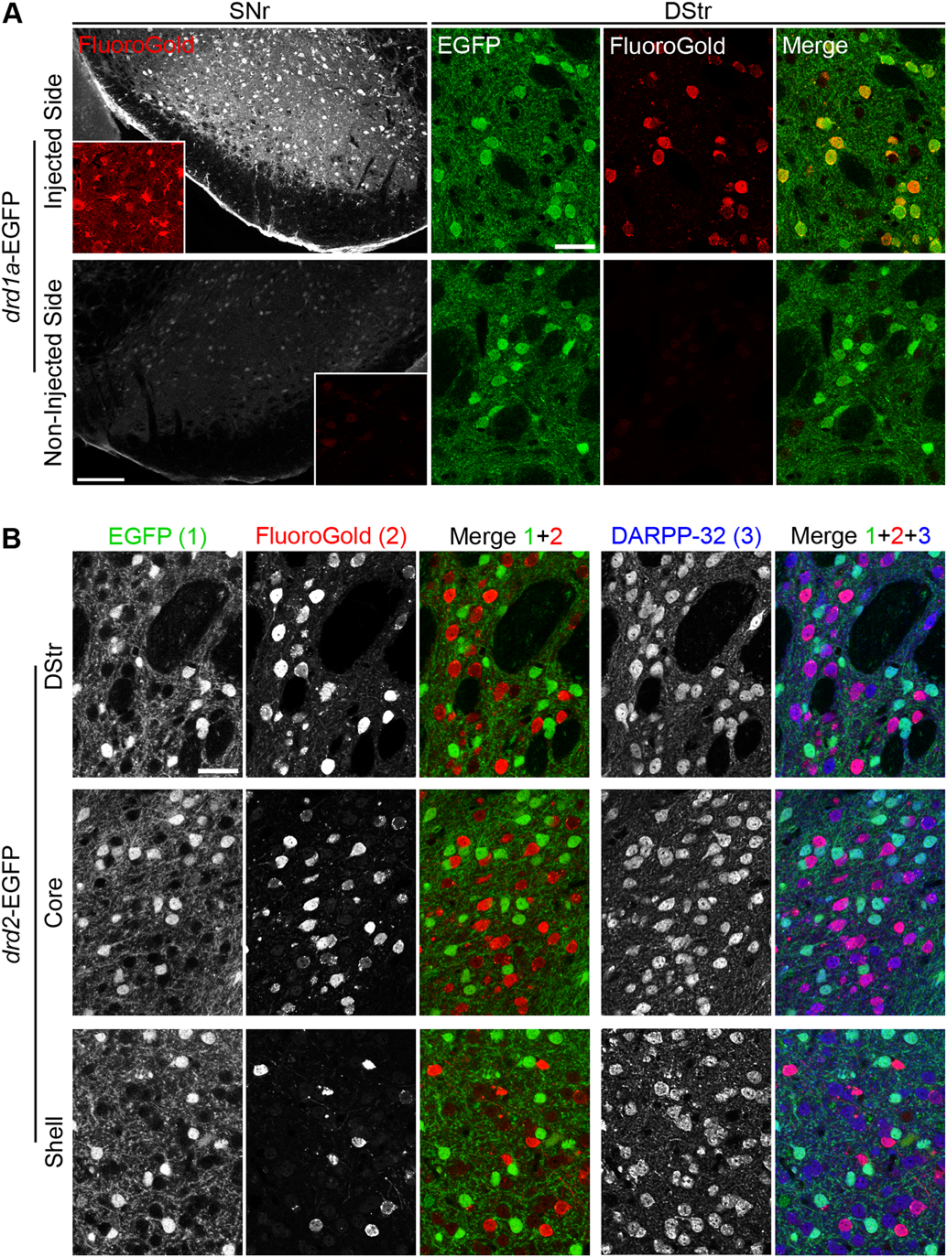
All striatonigral projection neurons express EGFP under the control of *drd1a* promoter. (A) Retrograde labeling of striatonigral neurons in *drd1a*-EGFP mice after unilateral FluoroGold injection in the substantia nigra pars reticulata (SNr). FluoroGold immunoreactivity (red) in the SNr (scale bar: 200 µm; insets: 4× magnification) and the dorsal striatum (DStr) of *drd1a*-EGFP mice in the hemisphere ipsilateral (injected side) or controlateral (non-injected side) to the injection site. In the injected side FluoroGold-immunoreactive neurons always contained EGFP fluorescence (i.e. expressed D1R). No FluoroGold-positive neurons were observed in the non-injected side. Scale bars: 40 µm. (B) The same experiment was carried out in *drd2*-EGFP mice. EGFP fluorescence (1) was detected together with FluoroGold immunoreactivity (2) and DARPP-32 immunoreactivity (3), in the DStr and NAc core (Core) and shell (Shell). FluoroGold-immunoreactive neurons did not contain EGFP in all striatal regions (Merge 1+2). All EGFP and FluoroGold-positive neurons were DARPP-32-immunoreactive MSNs (Merge 1+2+3). Images are single confocal sections. Scale bar: 40 µm.

**Figure 7:**
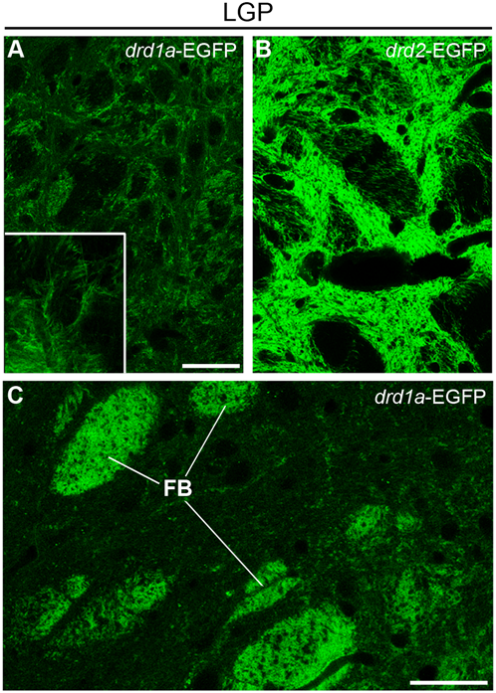
EGFP containing fibers in the lateral globus pallidus of *drd1a*-EGFP and *drd2*-EGFP mice. EGFP fluorescence was determined at low (A, B) and high (A inset and C) magnification in the LGP of *drd1a*-EGFP (A and C) and *drd2*-EGFP (B) mice. Scale bars: 40 µm. Inset: 4× stacked image. FB: fiber bundles presumably corresponding to striatonigral axons crossing the LGP in *drd1a*-EGFP mice. Note that outside of these bundles, a loose weave of intermingled fibers is spread across the LGP in *drd1a*-EGFP mice, possibly corresponding to sparse terminals, which are much less intense than those observed in *drd2*-EGFP mice.

Previous reports with BAC transgenic mice have shown that striatonigral neurons are essentially D1R-expressing neurons [12], [13], [19]. Because the present study lifted the uncertainty about the existence of non-EGFP-expressing MSNs in the BAC transgenic mice, we used these mice to reassess the nature of striatonigral neurons by retrograde labeling. FluoroGold, a retrograde tracer, was stereotactically injected in the SNr of *drd1a*-EGFP and *drd2*-EGFP mice. FluoroGold-immunoreactive neurons were detected in the striatum ipsilaterally to the injection side, but not on the contralateral side (Fig. 6A). In *drd1a*-EGFP mice, 100±0% of FluoroGold-positive neurons were labeled with EGFP, indicating that all striatonigral neurons express the D1R (298 neurons examined from two different mice, Fig. 6A). In *drd2*-EGFP mice, 99.3±0.6% of FluoroGold-immunoreactive neurons did not contain EGFP (566 neurons examined from 2 different mice, Fig. 6B). Thus only 4 out of 566 retrograde-labeled neurons (0.7%) expressed EGFP in *drd2*-EGFP neurons. Since in *drd1a*-EGFP mice FluoroGold was completely colocalized with EGFP, these results suggest that the few neurons which contained both EGFP and FluoroGold in *drd2*-EGFP mice expressed both D1 and D2 receptors. As expected, immunolabeling with DARPP-32 showed that both EGFP-neurons and FluoroGold-neurons were MSNs (Fig. 6B). Interestingly, detailed analysis of the LGP of *drd1a-*EGFP mice revealed a loose weave of intermingled fibers that could account for sparse terminals of striatonigral neurons targeting the pallidum (Fig. 7), in agreement with the basal fluorescent signal previously observed in the LGP of *drd1a*-EGFP mice [12], [13]. Such fibers might account for the few branched neurons projecting to both SNr and GP reported in rats [31], [32].

## Discussion

In the present study we show that nuclear staining with TO-PRO-3 provides a simple means for the identification of striatal neuronal populations in the absence of other markers. Appearance of chromatin is often casually used on electron micrographs to identify neuronal populations, but, to our knowledge, nuclear staining has not been exploited for systematic identification of striatal neurons. Comparison with DARPP-32 immunoreactivity, a well-established marker for MSNs [25], [28], [33], showed that these neurons have a distinct nuclear appearance following DNA staining, in comparison to other striatal cells. This appearance is sufficiently characteristic to allow their accurate identification. Other striatal neurons also have distinct nuclear staining patterns, which allow a good prediction of their nature. This simple method may be useful to identify striatal neurons in tissue sections and potentially in living tissue, using vital DNA fluorescent dyes. Since the intensity of TO-PRO-3 fluorescence reflects DNA concentration [34], [35], the intensely labeled areas presumably correspond to heterochromatin, which is more condensed than euchromatin. Thus, it is interesting to note that various neuronal types have distinct nuclear organizations, likely to reflect distinct different functional states [36], [37]. The relatively low abundance of condensed heterochromatin, reduced to 3–5 small clumps in MSNs may suggest that a larger repertoire of genes is accessible to transcription in these neurons than in other cell types. Similar nuclear appearances were observed for principal neurons in other brain regions such as pyramidal neurons of the cerebral cortex (data not shown). However, further studies are necessary to determine whether nuclear staining can be used for reliably identifying cell populations outside of the striatum, as we could predict.

Although it is clear that most striatal neurons are MSNs which project to the SNr or the LGP and express distinct proteins, the degree of segregation of these two populations has been intensely debated. Many studies in the last 20 years have attempted to quantify the proportion of MSNs expressing D1R, D2R or both by using various approaches [7], [38]–[40]. Converging evidence showed that striatonigral neurons express preferentially D1R [41], [42], dynorphin [38], and substance P [43]–[45], whereas striatopallidal neurons express preferentially D2R [46], enkephalin [38], [47], and A2A receptors [48], [49]. However, some results have suggested the existence of a fairly high degree of overlap between the expression of D1R and D2R [32]. The recent introduction of *drd1a*-EGFP and *drd2*-EGFP BAC transgenic mice allows an easy identification of D1R-expressing and D2R-expressing neurons [9], [12], [19]. The use of markers of various types of striatal neurons showed that EGFP was expressed in striatonigral MSNs of *drd1a*-EGFP mice, whereas striatopallidal MSNs and cholinergic interneurons were labeled in *drd2*-EGFP mice [11], [13]. These studies showed that approximately half of the MSNs were labeled with either probe, but they did not assess whether all the MSNs expressed one or the other. This information is critical, however, to estimate the number of MSNs expressing both receptors. Such measurement requires the expression of markers for D1R and D2R in the same animal. A recent paper elegantly addressed this question by using the *drd1a* promoter to drive the expression of tdTomato, a red fluorescent protein, in BAC transgenic mice [21]. However, when these authors crossed *drd1a*-tdTomato and *drd2*-EGFP mice, they estimated that up to 39% of MSNs did not express either fluorescent protein in the F1 progeny. The proportion of unlabeled neurons was even higher (50%) when they crossed *drd1a*-EGFP and *drd2*-EGFP mice. These results implied that either the fluorescent protein expression was too low to be detected in a large proportion of the neurons for some technical reason, or that a substantial number of MSNs did not express D1R or D2R. In any case, they casted doubt on the generality of the results obtained in BAC transgenic mice. They also implied that it was impossible to use the lack of expression of a marker as a means to identify a neuronal population. In light of our results, it appears that a major limitation of the study of Shuen *et al.* [21] was that the identification of neuronal populations in the striatum and distinction between neurons and glia was not convincingly done. In addition it is possible that some weakly fluorescent *drd1a*-EGFP expressing neurons were overlooked. Therefore the reported estimates may have been inaccurate. In the present study we carefully quantified striatal neuronal populations, using antibodies for NeuN, DARPP-32, various interneuron markers and nuclear staining patterns. We obtained convergent results with these various approaches which clearly showed that all MSNs expressed either D1R or D2R or both.

It is interesting to compare the quantifications of neurons carried out by various techniques. DARPP-32 antibodies were reported to label 96.4% of medium-sized neurons of the rat striatum (cell bodies 10–15 µm) [28]. In complete agreement with these results, we find that about 97% of neurons (NeuN-positive cells) were DARPP-32 positive. In our previous study [13] we estimated that 52% of the MSNs in the dorsal striatum, 53% in the NAc core and 47% in the shell expressed only D1R. These numbers are very similar to those obtained in the present study (51.1%, 51.4%, and 46.9%, respectively). Moreover, the demonstration that all MSNs express one or the other dopamine receptor allows us to conclude that the numbers previously calculated to estimate the proportion of neurons expressing both types of receptors (5%, 6%, and 17%, in the dorsal striatum, core, and shell, respectively, [13]) are not underestimations but are likely to reflect the real proportions. It should be kept in mind, however, that all these numbers are based on the expression of a reporter protein, EGFP, and not of receptors themselves.

Although striatonigral and striatopallidal pathways are distinct and mostly formed by different MSNs [38], anatomical and electrophysiological studies suggest the existence in rat and monkey of neurons which project to both SNr and GP [31], [32]. Having demonstrated that all MSNs express D1R or/and D2R, we used a retrograde neuronal tracer locally injected into the SNr to determine which population of neurons projected to the SNr. As expected, we found that all retrogradely labeled striatonigral neurons were EGFP-positive in *drd1a*-EGFP mice, whereas only very few (<1%) retrogradely labeled neurons expressed EGFP in *drd2*-EGFP mice. These observations demonstrate that striatonigral neurons all express D1R and that only a very tiny fraction may also express D2R. On the other hand, observations in our and other laboratories support the existence of a small fraction of D1R-neuron projections to the pallidum, since weak but noticeable staining is present in the LGP of *drd1a*-Cre, *chrm4*-EGFP and *drd1a*-EGFP mice [12], [13], [19]. Since striatonigral fibers cross the pallidum, it would be difficult to completely rule out the artefactual labeling of some of these fibers using retrograde staining. However, analysis of the LGP of *drd1a-*EGFP mice revealed a light interlace of fibers that could account for sparse terminals of striatonigral neurons in the pallidum. Such fibers might account for the few branched neurons projecting to both SNr and GP reported in rats [31], [32]. In contrast, our findings clearly show that the overwhelming majority of D2R-expressing neurons do not project to the SNr, in agreement with previous reports [31], [38].

The present study provides evidence that a simple nuclear staining method can be sufficient to identify MSNs, and also to recognize the various striatal interneurons with a good predictive value. The precise analysis of BAC transgenic mouse lines shows that 100% of the MSNs express D1R or D2R or in a minority of cases both. This shows that the use of a single transgenic line in combination with DARPP-32 staining or a nuclear marker is sufficient for the correct distinction of striatonigral and striatopallidal neurons in histological analysis.

## Supporting Information

Figure S1 Identification of striatal neurons based on their nuclear morphology. Images of TO-PRO-3-stained nuclei from 9 neurons of each striatal neural population identified with specific antibodies as in Fig. 3, were randomly numbered and mixed. The pictures were examined by four observers unaware of the identity of the neurons, who classified them into 5 categories according to the criteria of Table 1. Bars represent the means±SEM of correct identifications for each category.
(0.24 MB TIF)

Figure S2 Different levels of striatal EGFP fluorescence in drd1a-, drd2- and drd1a-EGFP/drd2- EGFP transgenic mice. (A–C) Confocal microscopy analysis at different photodetection voltages in striatal slices of drd1a-EGFP (A1, B1), drd2-EGFP (A2, B2) and drd1a-EGFP/drd2-EGFP mice (C). (A) At a low photomultiplier tube (PMT) voltage (621.3v), EGFP fluorescence appears weak in drd1a-EGFP mice, and striatal D1R neurons barely visualized (1). The same PMT voltage is sufficient for correct visualization of striatal D2R neurons in drd2-EGFP mice (2). Note the different degree of saturation of fluorescent neurons in drd1a-EGFP (1′) and drd2-EGFP mice (2′), as indicated by the blue coloring in a black-to-yellow look up table (LUT) color in each image (right). (B) A higher PMT detection voltage (727.4v) allows the correct visualization of striatal D1R neurons in drd1a-EGFP mice (1, 1′), whereas D2R neurons of drd2-EGFP mice appear saturated (2, 2′). (C) Striatal slices of drd1a-EGFP/drd2-EGFP double transgenic mice analyzed at a medium PMT voltage (641.0v), which allows the distinction of weakly labeled neurons (1, 1′) and strongly labeled neurons (2, 2′). Low-EGFP neurons are putative D1R neurons, whereas high-EGFP neurons are putative D2R neurons. Images are single confocal sections. Scale bars: 40 µm. For all panels the right picture is a 4× magnification of the area indicated in the left picture.
(7.59 MB TIF)

## References

[1] Groenewegen HJ (2003). The basal ganglia and motor control.. Neural Plast.

[2] Gurney K, Prescott TJ, Wickens JR, Redgrave P (2004). Computational models of the basal ganglia: from robots to membranes.. Trends Neurosci.

[3] Tepper JM, Bolam JP (2004). Functional diversity and specificity of neostriatal interneurons.. Curr Opin Neurobiol.

[4] Kawaguchi Y, Wilson CJ, Augood SJ, Emson PC (1995). Striatal interneurones: chemical, physiological and morphological characterization.. Trends Neurosci.

[5] Gerfen CR, Engber TM, Mahan LC, Susel Z, Chase TN (1990). D1 and D2 dopamine receptor-regulated gene expression of striatonigral and striatopallidal neurons.. Science.

[6] Fink JS, Weaver DR, Rivkees SA, Peterfreund RA, Pollack AE (1992). Molecular cloning of the rat A2 adenosine receptor: selective co-expression with D2 dopamine receptors in rat striatum.. Brain Res Mol Brain Res.

[7] Le Moine C, Bloch B (1995). D1 and D2 dopamine receptor gene expression in the rat striatum: sensitive cRNA probes demonstrate prominent segregation of D1 and D2 mRNAs in distinct neuronal populations of the dorsal and ventral striatum.. J Comp Neurol.

[8] Alexander GE, DeLong MR, Strick PL (1986). Parallel organization of functionally segregated circuits linking basal ganglia and cortex.. Annu Rev Neurosci.

[9] Gong S, Zheng C, Doughty ML, Losos K, Didkovsky N (2003). A gene expression atlas of the central nervous system based on bacterial artificial chromosomes.. Nature.

[10] Wang Z, Kai L, Day M, Ronesi J, Yin HH (2006). Dopaminergic control of corticostriatal long-term synaptic depression in medium spiny neurons is mediated by cholinergic interneurons.. Neuron.

[11] Lee KW, Kim Y, Kim AM, Helmin K, Nairn AC (2006). Cocaine-induced dendritic spine formation in D1 and D2 dopamine receptor-containing medium spiny neurons in nucleus accumbens.. Proc Natl Acad Sci U S A.

[12] Lobo MK, Karsten SL, Gray M, Geschwind DH, Yang XW (2006). FACS-array profiling of striatal projection neuron subtypes in juvenile and adult mouse brains.. Nat Neurosci.

[13] Bertran-Gonzalez J, Bosch C, Maroteaux M, Matamales M, Herve D (2008). Opposing patterns of signaling activation in dopamine D1 and D2 receptor-expressing striatal neurons in response to cocaine and haloperidol.. J Neurosci.

[14] Borgkvist A, Valjent E, Santini E, Herve D, Girault JA (2008). Delayed, context- and dopamine D1 receptor-dependent activation of ERK in morphine-sensitized mice.. Neuropharmacology.

[15] Gertler TS, Chan CS, Surmeier DJ (2008). Dichotomous anatomical properties of adult striatal medium spiny neurons.. J Neurosci.

[16] Shen W, Flajolet M, Greengard P, Surmeier DJ (2008). Dichotomous dopaminergic control of striatal synaptic plasticity.. Science.

[17] Gerfen CR, Paletzki R, Worley P (2008). Differences between dorsal and ventral striatum in Drd1a dopamine receptor coupling of dopamine- and cAMP-regulated phosphoprotein-32 to activation of extracellular signal-regulated kinase.. J Neurosci.

[18] Bateup HS, Svenningsson P, Kuroiwa M, Gong S, Nishi A (2008). Cell type-specific regulation of DARPP-32 phosphorylation by psychostimulant and antipsychotic drugs.. Nat Neurosci.

[19] Gong S, Doughty M, Harbaugh CR, Cummins A, Hatten ME (2007). Targeting Cre recombinase to specific neuron populations with bacterial artificial chromosome constructs.. J Neurosci.

[20] Kreitzer AC, Malenka RC (2007). Endocannabinoid-mediated rescue of striatal LTD and motor deficits in Parkinson's disease models.. Nature.

[21] Shuen JA, Chen M, Gloss B, Calakos N (2008). Drd1a-tdTomato BAC transgenic mice for simultaneous visualization of medium spiny neurons in the direct and indirect pathways of the basal ganglia.. J Neurosci.

[22] Paxinos GF, KBJ (2001). The Mouse Brain in stereotaxic coordinates..

[23] Suzuki T, Fujikura K, Higashiyama T, Takata K (1997). DNA staining for fluorescence and laser confocal microscopy.. J Histochem Cytochem.

[24] Bink K, Walch A, Feuchtinger A, Eisenmann H, Hutzler P (2001). TO-PRO-3 is an optimal fluorescent dye for nuclear counterstaining in dual-colour FISH on paraffin sections.. Histochem Cell Biol.

[25] Ouimet CC, Greengard P (1990). Distribution of DARPP-32 in the basal ganglia: an electron microscopic study.. J Neurocytol.

[26] Stipanovich A, Valjent E, Matamales M, Nishi A, Ahn JH (2008). A phosphatase cascade by which rewarding stimuli control nucleosomal response.. Nature.

[27] Mullen RJ, Buck CR, Smith AM (1992). NeuN, a neuronal specific nuclear protein in vertebrates.. Development.

[28] Ouimet CC, Langley-Gullion KC, Greengard P (1998). Quantitative immunocytochemistry of DARPP-32-expressing neurons in the rat caudatoputamen.. Brain Res.

[29] Takagi H, Somogyi P, Somogyi J, Smith AD (1983). Fine structural studies on a type of somatostatin-immunoreactive neuron and its synaptic connections in the rat neostriatum: a correlated light and electron microscopic study.. J Comp Neurol.

[30] Le Moine C, Tison F, Bloch B (1990). D2 dopamine receptor gene expression by cholinergic neurons in the rat striatum.. Neurosci Lett.

[31] Kawaguchi Y, Wilson CJ, Emson PC (1990). Projection subtypes of rat neostriatal matrix cells revealed by intracellular injection of biocytin.. J Neurosci.

[32] Levesque M, Bedard A, Cossette M, Parent A (2003). Novel aspects of the chemical anatomy of the striatum and its efferents projections.. J Chem Neuroanat.

[33] Ouimet CC, Miller PE, Hemmings HC,, Walaas SI, Greengard P (1984). DARPP-32, a dopamine- and adenosine 3′:5′-monophosphate-regulated phosphoprotein enriched in dopamine-innervated brain regions. III. Immunocytochemical localization.. J Neurosci.

[34] Stoica BA, Movsesyan VA, Knoblach SM, Faden AI (2005). Ceramide induces neuronal apoptosis through mitogen-activated protein kinases and causes release of multiple mitochondrial proteins.. Mol Cell Neurosci.

[35] Oomman S, Strahlendorf H, Finckbone V, Strahlendorf J (2005). Non-lethal active caspase-3 expression in Bergmann glia of postnatal rat cerebellum.. Brain Res Dev Brain Res.

[36] Bishop GA, Chang HT, Kitai ST (1982). Morphological and physiological properties of neostriatal neurons: an intracellular horseradish peroxidase study in the rat.. Neuroscience.

[37] DiFiglia M, Carey J (1986). Large neurons in the primate neostriatum examined with the combined Golgi-electron microscopic method.. J Comp Neurol.

[38] Gerfen CR, Young WS, (1988). Distribution of striatonigral and striatopallidal peptidergic neurons in both patch and matrix compartments: an in situ hybridization histochemistry and fluorescent retrograde tracing study.. Brain Res.

[39] Yung KK, Bolam JP, Smith AD, Hersch SM, Ciliax BJ (1995). Immunocytochemical localization of D1 and D2 dopamine receptors in the basal ganglia of the rat: light and electron microscopy.. Neuroscience.

[40] Surmeier DJ, Song WJ, Yan Z (1996). Coordinated expression of dopamine receptors in neostriatal medium spiny neurons.. J Neurosci.

[41] Herrera-Marschitz M, Ungerstedt U (1984). Evidence that striatal efferents relate to different dopamine receptors.. Brain Res.

[42] Beckstead RM (1988). Association of dopamine D1 and D2 receptors with specific cellular elements in the basal ganglia of the cat: the uneven topography of dopamine receptors in the striatum is determined by intrinsic striatal cells, not nigrostriatal axons.. Neuroscience.

[43] Mroz EA, Brownstein MJ, Leeman SE (1977). Evidence for substance P in the striato-nigral tract.. Brain Res.

[44] Brownstein MJ, Mroz EA, Tappaz ML, Leeman SE (1977). On the origin of substance P and glutamic acid decarboxylase (GAD) in the substantia nigra.. Brain Res.

[45] Hong JS, Yang HY, Racagni G, Costa E (1977). Projections of substance P containing neurons from neostriatum to substantia nigra.. Brain Res.

[46] Le Moine C, Normand E, Guitteny AF, Fouque B, Teoule R (1990). Dopamine receptor gene expression by enkephalin neurons in rat forebrain.. Proc Natl Acad Sci U S A.

[47] Hong JS, Yang HY, Costa E (1977). On the location of methionine enkephalin neurons in rat striatum.. Neuropharmacology.

[48] Schiffmann SN, Jacobs O, Vanderhaeghen JJ (1991). Striatal restricted adenosine A2 receptor (RDC8) is expressed by enkephalin but not by substance P neurons: an in situ hybridization histochemistry study.. J Neurochem.

[49] Augood SJ, Emson PC (1994). Adenosine A2a receptor mRNA is expressed by enkephalin cells but not by somatostatin cells in rat striatum: a co-expression study.. Brain Res Mol Brain Res.

