# Supplementary figures and images for: Striatal Medium-Sized Spiny Neurons: Identification by Nuclear Staining and Study of Neuronal Subpopulations in BAC Transgenic Mice
Source: PLoS One. 2009 Mar 10;4(3):e4770. doi: 10.1371/journal.pone.0004770 (PMC2651623; doi:10.1371/journal.pone.0004770)

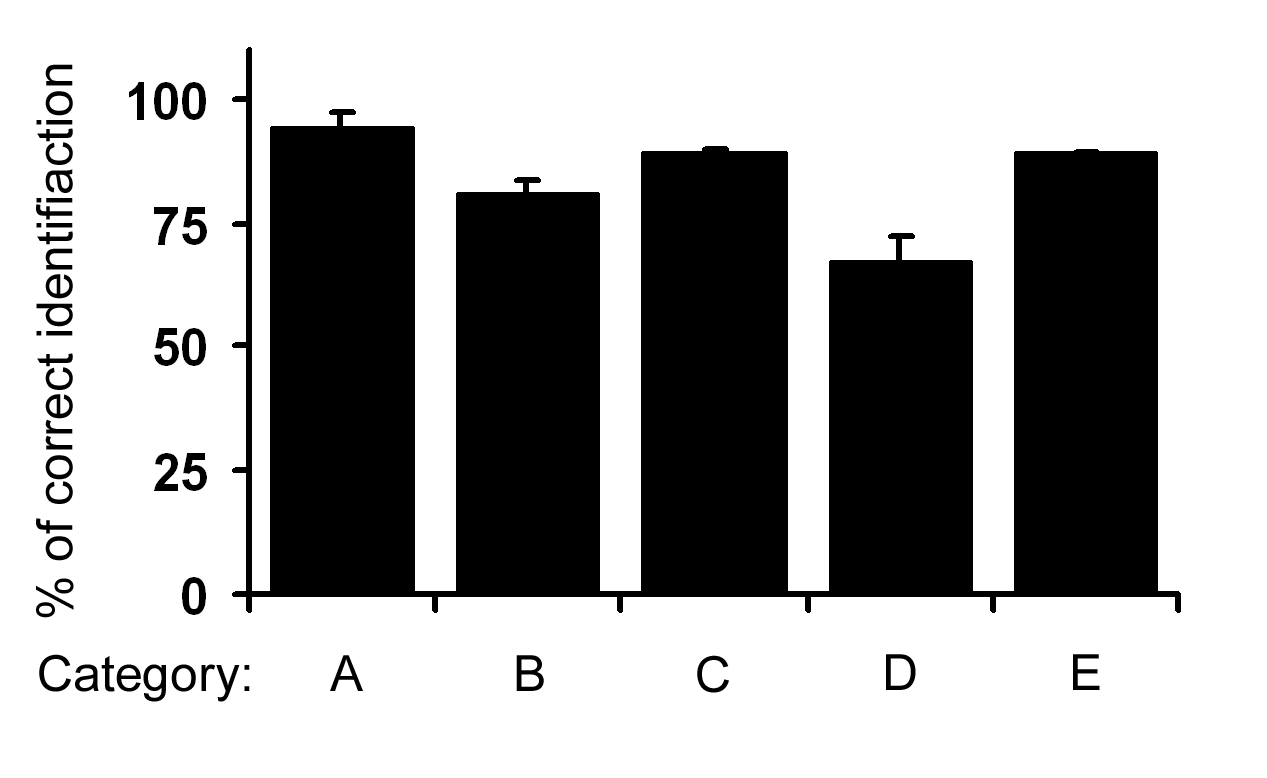

Supplement: Figure S1 — Identification of striatal neurons based on their nuclear morphology. Images of TO-PRO-3-stained nuclei from 9 neurons of each striatal neural population identified with specific antibodies as in Fig. 3, were randomly numbered and mixed. The pictures were examined by four observers unaware of the identity of the neurons, who classified them into 5 categories according to the criteria of Table 1. Bars represent the means±SEM of correct identifications for each category. (0.24 MB TIF) [file pone.0004770.s001.tif]

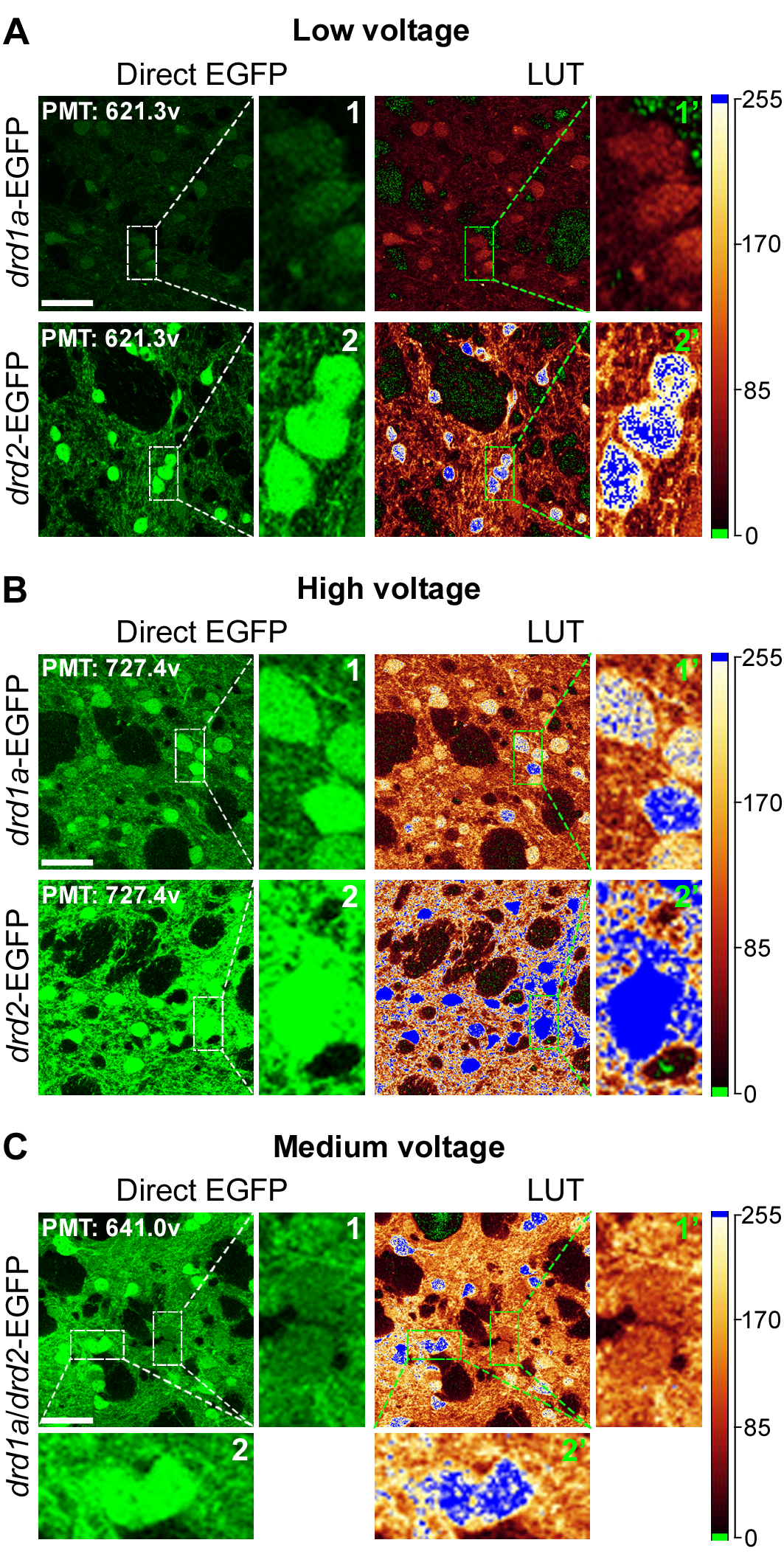

Supplement: Figure S2 — Different levels of striatal EGFP fluorescence in drd1a-, drd2- and drd1a-EGFP/drd2- EGFP transgenic mice. (A–C) Confocal microscopy analysis at different photodetection voltages in striatal slices of drd1a-EGFP (A1, B1), drd2-EGFP (A2, B2) and drd1a-EGFP/drd2-EGFP mice (C). (A) At a low photomultiplier tube (PMT) voltage (621.3v), EGFP fluorescence appears weak in drd1a-EGFP mice, and striatal D1R neurons barely visualized (1). The same PMT voltage is sufficient for correct visualization of striatal D2R neurons in drd2-EGFP mice (2). Note the different degree of saturation of fluorescent neurons in drd1a-EGFP (1′) and drd2-EGFP mice (2′), as indicated by the blue coloring in a black-to-yellow look up table (LUT) color in each image (right). (B) A higher PMT detection voltage (727.4v) allows the correct visualization of striatal D1R neurons in drd1a-EGFP mice (1, 1′), whereas D2R neurons of drd2-EGFP mice appear saturated (2, 2′). (C) Striatal slices of drd1a-EGFP/drd2-EGFP double transgenic mice analyzed at a medium PMT voltage (641.0v), which allows the distinction of weakly labeled neurons (1, 1′) and strongly labeled neurons (2, 2′). Low-EGFP neurons are putative D1R neurons, whereas high-EGFP neurons are putative D2R neurons. Images are single confocal sections. Scale bars: 40 µm. For all panels the right picture is a 4× magnification of the area indicated in the left picture. (7.59 MB TIF) [file pone.0004770.s002.tif]
